# Vaccines and myocardial injury in patients hospitalized for COVID-19 infection: the CardioCOVID-Gemelli study

**DOI:** 10.1093/ehjqcco/qcae016

**Published:** 2024-02-27

**Authors:** Rocco Antonio Montone, Riccardo Rinaldi, Carlotta Masciocchi, Livia Lilli, Andrea Damiani, Giulia La Vecchia, Giulia Iannaccone, Mattia Basile, Carmine Salzillo, Andrea Caffè, Alice Bonanni, Gennaro De Pascale, Domenico Luca Grieco, Eloisa Sofia Tanzarella, Danilo Buonsenso, Rita Murri, Massimo Fantoni, Giovanna Liuzzo, Tommaso Sanna, Luca Richeldi, Maurizio Sanguinetti, Massimo Massetti, Carlo Trani, Yamume Tshomba, Antonio Gasbarrini, Vincenzo Valentini, Massimo Antonelli, Filippo Crea

**Affiliations:** Department of Cardiovascular Sciences, Fondazione Policlinico Universitario A. Gemelli IRCCS, 00168 Rome, Italy; Department of Cardiovascular and Pulmonary Sciences, Catholic University of the Sacred Heart, 00168 Rome, Italy; Real World Data Facility, Gemelli Generator, Fondazione Policlinico Universitario Agostino Gemelli IRCCS, 00168 Rome, Italy; Real World Data Facility, Gemelli Generator, Fondazione Policlinico Universitario Agostino Gemelli IRCCS, 00168 Rome, Italy; Dipartimento di Scienze di Laboratorio e Infettivologiche, Fondazione Policlinico Universitario A. Gemelli IRCCS, 00168 Roma, Italy; Department of Cardiovascular and Pulmonary Sciences, Catholic University of the Sacred Heart, 00168 Rome, Italy; Department of Cardiovascular and Pulmonary Sciences, Catholic University of the Sacred Heart, 00168 Rome, Italy; Department of Cardiovascular and Pulmonary Sciences, Catholic University of the Sacred Heart, 00168 Rome, Italy; Department of Cardiovascular and Pulmonary Sciences, Catholic University of the Sacred Heart, 00168 Rome, Italy; Department of Cardiovascular and Pulmonary Sciences, Catholic University of the Sacred Heart, 00168 Rome, Italy; Department of Cardiovascular Sciences, Fondazione Policlinico Universitario A. Gemelli IRCCS, 00168 Rome, Italy; Department of Emergency, Intensive Care Medicine and Anaesthesia, Fondazione Policlinico Universitario A. Gemelli IRCCS, 00168 Rome, Italy; Istituto di Anestesiologia e Rianimazione, Università Cattolica del Sacro Cuore, 00168 Rome, Italy; Department of Emergency, Intensive Care Medicine and Anaesthesia, Fondazione Policlinico Universitario A. Gemelli IRCCS, 00168 Rome, Italy; Department of Emergency, Intensive Care Medicine and Anaesthesia, Fondazione Policlinico Universitario A. Gemelli IRCCS, 00168 Rome, Italy; Department of Women's Health, Child Health and Public Health Sciences, Fondazione Policlinico Universitario A. Gemelli IRCCS, 00168 Rome, Italy; Dipartimento di Scienze di Laboratorio e Infettivologiche, Fondazione Policlinico Universitario A. Gemelli IRCCS, 00168 Roma, Italy; Clinic of Infectious Diseases, Catholic University of the Sacred Heart, 00168 Rome, Italy; Dipartimento di Scienze di Laboratorio e Infettivologiche, Fondazione Policlinico Universitario A. Gemelli IRCCS, 00168 Roma, Italy; Clinic of Infectious Diseases, Catholic University of the Sacred Heart, 00168 Rome, Italy; Department of Cardiovascular Sciences, Fondazione Policlinico Universitario A. Gemelli IRCCS, 00168 Rome, Italy; Department of Cardiovascular and Pulmonary Sciences, Catholic University of the Sacred Heart, 00168 Rome, Italy; Department of Cardiovascular Sciences, Fondazione Policlinico Universitario A. Gemelli IRCCS, 00168 Rome, Italy; Department of Cardiovascular and Pulmonary Sciences, Catholic University of the Sacred Heart, 00168 Rome, Italy; Division of Pulmonary Medicine, Fondazione Policlinico Universitario Agostino Gemelli IRCCS, Università Cattolica del Sacro Cuore, 00168 Rome, Italy; Department of Basic Biotechnological Sciences, Intensive and Perioperative Clinics, Catholic University of the Sacred Heart, 00168 Rome, Italy; Department of Cardiovascular Sciences, Fondazione Policlinico Universitario A. Gemelli IRCCS, 00168 Rome, Italy; Department of Cardiovascular and Pulmonary Sciences, Catholic University of the Sacred Heart, 00168 Rome, Italy; Department of Cardiovascular Sciences, Fondazione Policlinico Universitario A. Gemelli IRCCS, 00168 Rome, Italy; Department of Cardiovascular and Pulmonary Sciences, Catholic University of the Sacred Heart, 00168 Rome, Italy; Department of Cardiovascular Sciences, Fondazione Policlinico Universitario A. Gemelli IRCCS, 00168 Rome, Italy; Department of Cardiovascular and Pulmonary Sciences, Catholic University of the Sacred Heart, 00168 Rome, Italy; Department of Medical and Surgical Sciences, Fondazione Policlinico Universitario A. Gemelli IRCCS, 00168 Rome, Italy; Department of Translational Medicine and Surgery, Catholic University of the Sacred Heart, 00168 Rome, Italy; Department of Diagnostic Imaging, Radiotherapy, Oncology and Hematology, Fondazione Policlinico Universitario A. Gemelli IRCCS, 00168 Rome, Italy; Department of Radiological and Hematological Sciences, Catholic University of the Sacred Heart, 00168 Rome, Italy; Department of Emergency, Intensive Care Medicine and Anaesthesia, Fondazione Policlinico Universitario A. Gemelli IRCCS, 00168 Rome, Italy; Istituto di Anestesiologia e Rianimazione, Università Cattolica del Sacro Cuore, 00168 Rome, Italy; Department of Cardiovascular Sciences, Fondazione Policlinico Universitario A. Gemelli IRCCS, 00168 Rome, Italy; Department of Cardiovascular and Pulmonary Sciences, Catholic University of the Sacred Heart, 00168 Rome, Italy

**Keywords:** Myocardial injury, SARS-CoV-2, COVID-19, Vaccines, Clinical predictors

## Abstract

**Background:**

Myocardial injury is prevalent among patients hospitalized for COVID-19. However, the role of COVID-19 vaccines in modifying the risk of myocardial injury is unknown.

**Aims:**

To assess the role of vaccines in modifying the risk of myocardial injury in COVID-19.

**Methods and results:**

We enrolled COVID-19 patients admitted from March 2021 to February 2022 with known vaccination status and ≥1 assessment of hs-cTnI within 30 days from the admission. The primary endpoint was the occurrence of myocardial injury (hs-cTnI levels >99th percentile upper reference limit). A total of 1019 patients were included (mean age: 67.7 ± 14.8 years, 60.8% male, and 34.5% vaccinated against COVID-19). Myocardial injury occurred in 145 (14.2%) patients. At multivariate logistic regression analysis, advanced age, chronic kidney disease, and hypertension, but not vaccination status, were independent predictors of myocardial injury. In the analysis according to age tertiles distribution, myocardial injury occurred more frequently in the III tertile (≥76 years) compared with other tertiles (I tertile: ≤60 years; II tertile: 61–75 years) (*P* < 0.001). Moreover, in the III tertile, vaccination was protective against myocardial injury [odds ratio (OR): 0.57, 95% confidence interval (CI): 0.34–0.94; *P* = 0.03], while a previous history of coronary artery disease was an independent positive predictor. In contrast, in the I tertile, chronic kidney disease (OR: 6.94, 95% CI: 1.31–36.79, *P* = 0.02) and vaccination (OR: 4.44, 95% CI: 1.28–15.34, *P* = 0.02) were independent positive predictors of myocardial injury.

**Conclusion:**

In patients ≥76 years, COVID-19 vaccines were protective for the occurrence of myocardial injury, while in patients ≤60 years, myocardial injury was associated with previous COVID-19 vaccination. Further studies are warranted to clarify the underlying mechanisms.

## Graphical Abstract

**Graphical Abstract:**
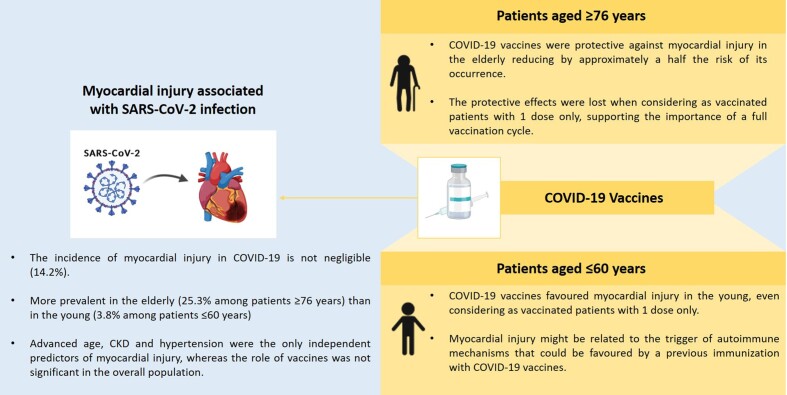

Key Learning Points What is already known:

- Myocardial injury is prevalent among patients hospitalized for COVID-19 and associated with worse outcome.
- COVID-19 vaccines demonstrated a high efficacy in protecting against SARS-CoV-2 infection.
- The role of COVID-19 vaccines in modifying the risk of myocardial injury associated with SARS-CoV-2 infection is unknown.

What this study adds:

- The incidence of myocardial injury in patients hospitalized for COVID-19 is not negligible and varies according to age.
- Advanced age, chronic kidney disease, and hypertension are independent predictors of myocardial injury, whereas the role of vaccines in modifying the risk of myocardial injury is not significant.
- The effect of vaccine on the risk of myocardial injury may vary across different age strata, being protective in the elderly, while favouring its occurrence in the young. Further studies are needed to clarify the mechanisms responsible for the interaction between age and vaccination in determining COVID-19–associated myocardial injury.

## Introduction

Coronavirus disease-2019 (COVID-19) is a global pandemic caused by the novel severe acute respiratory syndrome coronavirus 2 (SARS-CoV-2) that is resulting in substantial morbidity and mortality worldwide.^1^ Even though the clinical manifestations of COVID-19 mainly involve the respiratory tract, a significant proportion of patients may demonstrate biomarker evidence of myocardial injury (defined as increased cardiac troponin levels) that is associated with worse outcomes.^2–5^ Advanced age and a previous history of cardiovascular (CV) diseases, including coronary artery disease (CAD), atrial fibrillation (AF), hypertension, and heart failure (HF), are likely to increase the risk of myocardial injury associated with SARS-CoV-2 infection but, to date, data are still scarce and sometimes inconsistent.^6–11^ Moreover, the pathogenesis of myocardial injury in COVID-19 is still debated, with proposed mechanisms that include direct cytokine-mediated damage, oxygen supply–demand imbalance, ischaemic injury from microvascular thrombi formation, direct viral invasion of the myocardium, and acute coronary syndrome from acute inflammation-triggered destabilization of atherosclerotic plaques.^1,12,13^

Recently, COVID-19 vaccines have been introduced and demonstrated a high efficacy in protecting against SARS-CoV-2 infection^14^ but they have also been associated with an increased, although low, risk of myocardial injury due to myocarditis, especially in young individuals receiving messenger RNA (mRNA)-based vaccines.^15^

Of note, despite initial evidence suggesting a role for SARS-CoV-2 infection in inducing myocardial injury,^2–5^ there are no studies to date evaluating the incidence and predictors of myocardial injury after the introduction of COVID-19 vaccines and, as a consequence, the role of a previous immunization with COVID-19 vaccines on the risk of myocardial injury associated with SARS-CoV-2 infection is unknown.

Therefore, the aim of the ‘Characterization and prognostic relevance of myocardial injury in patients with coronavirus disease-2019’ (CardioCOVID-Gemelli) study was to assess the incidence and predictors of myocardial injury in a large cohort of patients hospitalized for COVID-19 infection since the introduction of vaccines, aiming in particular to evaluate the role of COVID-19 vaccines in modifying the risk of myocardial injury associated with SARS-CoV-2 infection.

## Study design and methods

### Study population

We conducted a retrospective analysis of the COVID-19 Data Mart, a prospectively enrolling real-time archive of structured and unstructured data, refreshed on a daily basis, and including all patients admitted for COVID-19 at Fondazione Policlinico A. Gemelli IRCCS Hospital, a main hub for COVID-19 patients in Rome, Italy.^16^

In particular, the study cohort included all patients admitted from March 2021 to January 2022 who were ≥18 years and had a positive nasopharyngeal swab for SARS-CoV-2 within the first 48 h of admission, at least one assessment of high-sensitivity cardiac troponin I (hs-cTnI) within the first 30 days from the admission, and known vaccination status for COVID-19 (see Supplementary material online, Appendix *Figure S1*). The diagnosis of SARS-CoV-2 infection was considered positive when the reverse transcription polymerase chain reaction (PCR) of the SARS-CoV-2 assay was detected from a nasopharyngeal swab.

### Data collection

Variables collected included demographics, comorbidities, vital signs, and laboratory measurements, as well as vaccination status for COVID-19 and clinical outcome during the index hospitalization (see Supplementary Appendix online for further details). Regarding vaccination status for COVID-19, patients were considered vaccinated either 2 weeks after they received their second dose in a two-dose series (e.g. Pfizer/BioNTech Comirnaty, Spikevax/Moderna, or Vaxzevria/AstraZeneca vaccines), or 2 weeks after their first dose for single-dose vaccines (e.g. Jcovden/Janssen vaccine), or 2 weeks after their second dose when the specific type of COVID-19 vaccines was not known. While concerning ECG abnormalities, new Q-wave appearance, ST elevation or depression, or T-wave abnormalities were collected. All data were extracted from the electronic medical records (EMR) of all patients. To obtain structural information from unstructured texts (such as clinical diary, radiology reports, etc.), Natural Language Processing (NLP) algorithms were applied, based on text mining procedures such as: sentence/word tokenization; rule-based approach supported by annotations defined by the clinical subject matter experts, and using semantic/syntactic corrections where necessary.^17^ Data regarding vaccination status were extracted from EMR or obtained through telephonic interview or medical visit. The study protocol complied with the Declaration of Helsinki and the study was approved by the Ethics Committee of the Fondazione Policlinico Gemelli (Comitato Etico Policlinico Gemelli; ID 4923). Written informed consent was waived because of the rapid emergence of this infectious disease.

### Endpoint

The primary endpoint was the occurrence of myocardial injury, defined as the elevation of hs-cTnI levels >99th percentile upper reference limit (>56 ng/L for a normal population).

### Statistical analysis

Continuous variables were expressed as mean ± standard deviation (SD) or as median and inter-quartile range, respectively, in the case they were normally or not normally distributed, and data were compared using Student's *t*-test or Mann–Whitney *U* test, as appropriate. Quantitative data distribution was assessed using the Shapiro–Wilk test. Categorical data were reported as absolute and relative percentage frequencies and between-group differences were evaluated using the χ^2^ test or Fisher’s exact test, as appropriate. A value of *P* < 0.05 was considered significant. A multivariable logistic regression analysis for the occurrence of myocardial injury was performed including all variables with a *P* value < 0.05 at univariate analysis. Results were expressed as odds ratio (OR) with 95% confidence interval (CI). Furthermore, the overall population was categorized into three tertiles according to age distribution (I tertile: ≤60 years; II tertile: 61–75 years; and III tertile: ≥76 years) and a multivariable logistic regression analysis for the occurrence of myocardial injury was performed in each tertile to assess the presence of different predictors across age strata. A two-tailed analysis was performed and a *P* value < 0.05 was considered as statistically significant. Finally, to explore the effect of a single-dose administration of COVID-19 vaccines, we also performed a sensitivity analysis for myocardial injury in the overall population and according to age strata considering as vaccinated patients who received one dose of any type of vaccines. All analyses were performed using SPSS version 21 (SPSS Inc., Chicago, IL, USA).

## Results

### Baseline characteristics of study population

A total of 1019 patients [mean age: 67.7 ± 14.8 years; 620 (60.8%) men] were included in the analysis. Myocardial injury occurred in 145 (14.2%) patients.

Patients with myocardial injury, compared with those without, were older (77.4 ± 13.1 vs. 66.1 ± 14.5 years, *P* < 0.001); had a higher prevalence of chronic kidney disease (CKD) [30 (20.7%) vs. 62 (7.1%), *P* < 0.001], type 2 diabetes mellitus (T2DM) [36 (24.8%) vs. 139 (15.9%), *P* = 0.008], hypertension [95 (65.5%) vs. 394 (45.1%), *P* < 0.001], history of HF [21 (14.5%) vs. 42 (4.8%), *P* < 0.001], chronic obstructive pulmonary disease (COPD) [30 (20.7%) vs. 103 (11.8%), *P* = 0.003], paroxysmal/persistent AF [37 (25.5%) vs. 83 (9.5%), *P* < 0.001], and history of CAD [38 (26.2%) vs. 104 (11.9%), *P* < 0.001]; and were more frequently vaccinated against COVID-19 [61 (42.1%) vs. 291 (33.3%), *P* = 0.040] without significant differences according to the type of vaccine, time from the last dose of vaccines to troponin assessment, and time from last dose of vaccine to positive PCR test for SARS-CoV-2 (all *P* > 0.05).

Moreover, patients with myocardial injury, compared with those without, had lower levels of haemoglobin (12.6 ± 2.3 vs. 13.7 ± 2.0 g/dL, *P* < 0.001), as well as higher levels of white blood cell (WBC) count (10.2 ± 8.3 vs. 7.7 ± 5.8 × 10^9^/L, *P* < 0.001), serum creatinine (1.6 ± 1.8 vs. 1.1 ± 1.1 mg/dL, *P* < 0.001), hs-cTnI at admission (1770.1 ± 8887.2 vs. 13.6 ± 12.3 ng/L, *P* < 0.001) and peak value during the hospitalization (3095.4 ± 11495.4 vs. 22.2 ± 110.2 ng/L, *P* < 0.001), D-dimer (4772.8 ± 7899.2 vs. 1829.2 ± 4111.0 ng/mL, *P* < 0.001), N-terminal prohormone of brain natriuretic peptide (NT-proBNP) (8653.8 ± 14184.4 vs. 1227.2 ± 3482.8 pg/mL, *P* < 0.001), C-reactive protein (100.3 ± 74.2 vs. 71.8 ± 63.4 mg/L, *P* < 0.001), and procalcitonin (2.0 ± 7.8 vs. 0.4 ± 1.9 ng/mL, *P* < 0.001).

Additionally, patients with myocardial injury, compared with those without, had a longer length of hospitalization (19.2 ± 17.9 vs. 15.6 ± 12.2 days, *P* = 0.002), higher positive haemocultures results [45 (31%) vs. 97 (11.1%), *P* < 0.001], a higher need for mechanic ventilation [35 (24.1%) vs. 88 (10.1%), *P* < 0.001] and admission to intensive care unit (ICU) [58 (40.0%) vs. 154 (17.6%), *P* < 0.001], and a higher prevalence of in-hospital deaths [54 (37.2%) vs. 68 (7.8%), *P* < 0.001]. Baseline characteristics of the overall population and according to the presence or absence of myocardial injury are shown in *Table 1*. Baseline characteristics of the overall population and according to the time waves of COVID-19 infection are shown in Supplementary material online, Appendix *Table S1*.

**Table 1:** Baseline characteristics of the overall population and according to the presence or absence of myocardial injury

Characteristics	Overall population (*n* = 1019)	Patients with myocardial injury (*n* = 145)	Patients with no myocardial injury (*n* = 874)	*P*
Clinical characteristics				
Age [mean ± SD]	67.7 ± 14.8	77.4 ± 13.1	66.1 ± 14.5	**<0.001**
Male sex [*n*, (%)]	620 (60.8)	93 (64.1)	527 (60.3)	0.380
CKD (eGFR <60 mL/min per 1.73 m^2^) [*n*, (%)]	92 (9.0)	30 (20.7)	62 (7.1)	**<0.001**
History of cancer [*n*, (%)]	148 (14.5)	22 (15.2)	126 (14.4)	0.811
T2DM [*n*, (%)]	175 (17.2)	36 (24.8)	139 (15.9)	**0.008**
Hypertension [*n*, (%)]	489 (48.0)	95 (65.5)	394 (45.1)	**<0.001**
History of HF [*n*, (%)]	63 (6.2)	21 (14.5)	42 (4.8)	**<0.001**
Chronic obstructive pulmonary disease [*n*, (%)]	133 (13.1)	30 (20.7)	103 (11.8)	**0.003**
Asthma [*n*, (%)]	40 (3.9)	5 (3.4)	35 (4.0)	1.000
Paroxysmal/persistent AF [*n*, (%)]	120 (11.8)	37 (25.5)	83 (9.5)	**<0.001**
History of CAD [*n*, (%)]	142 (13.9)	38 (26.2)	104 (11.9)	**<0.001**
DBP at admission (mmHg) [mean ± SD]	79.0 ± 12.4	76.7 ± 13.5	79.4 ± 12.2	**0.028**
SBP at admission (mmHg) [mean ± SD]	132.6 ± 20.6	130.2 ± 23.0	132.9 ± 20.3	0.189
Vaccination status				
Vaccinated (≥2 doses^a^) against COVID-19 [*n*, (%)]	352 (34.5)	61 (42.1)	291 (33.3)	**0.040**
Total COVID-19 vaccine doses administered [*n*, (%)]				**0.021**
Not vaccinated [*n*, (%)]	601 (59.0)	70 (48.3)	531 (60.8)	
1 dose [*n*, (%)]	66 (6.5)	14 (9.7)	52 (5.9)	
2 doses^a^ [*n*, (%)]	286 (28.1)	47 (32.4)	239 (27.3)	
3 doses [*n*, (%)]	66 (6.5)	14 (9.7)	52 (5.9)	
Type of COVID-19 vaccine [*n*, (%)]				0.053
Pfizer/BioNTech Comirnaty [*n*, (%)]	184 (18.1)	35 (24.1)	149 (17.0)	
Vaxzevria/AstraZeneca [*n*, (%)]	27 (2.6)	1 (0.7)	26 (3.0)	
Spikevax/Moderna [*n*, (%)]	11 (1.1)	3 (2.1)	8 (0.9)	
Jcovden/Janssen [*n*, (%)]	5 (0.5)	0 (0.0)	5 (0.6)	
Missing data [*n*, (%)]	125 (12.3)	22 (15.2)	103 (11.8)	
Time from last dose of vaccine to troponin assessment (days) [mean ± SD]	122.9 ± 84.7	114.7 ± 86.0	124.6 ± 84.5	0.491
Time from last dose of vaccine to positive PCR test for SARS-CoV-2 (days) [mean ± SD]	121.0 ± 84.5	113.1 ± 86.2	122.6 ± 84.3	0.507
Laboratory data				
Hb (g/dL) [mean ± SD]	13.5 ± 2.1	12.6 ± 2.3	13.7 ± 2.0	**<0.001**
WBC (×10^9^/L) [mean ± SD]	8.0 ± 6.3	10.2 ± 8.3	7.7 ± 5.8	**<0.001**
Serum creatinine on admission (mg/dL) [mean ± SD]	1.2 ± 1.3	1.6 ± 1.8	1.1 ± 1.1	**<0.001**
Total protein (g/L) [mean ± SD]	66.2 ± 6.6	64.4 ± 7.3	66.5 ± 6.5	**0.001**
Antithrombin (%) [mean ± SD]	101.4 ± 17.5	92.6 ± 16.3	102.8 ± 17.3	**<0.001**
hs-cTnI at admission (ng/L) [mean ± SD]	263.5 ± 3398.5	1770.1 ± 8887.2	13.6 ± 12.3	**<0.001**
hs-cTnI peak during index hospitalization (ng/L) [mean ± SD]	459.5 ± 4456.1	3095.4 ± 11495.4	22.2 ± 110.2	**<0.001**
LDH (UI/L) [mean ± SD]	406.6 ± 297.0	470.3 ± 528.0	396.1 ± 237.3	**0.005**
D-dimer (ng/mL) [mean ± SD]	2238.9 ± 4920.8	4773.8 ± 7899.2	1829.2 ± 4111.0	**<0.001**
Fibrinogen (mg/dL) [mean ± SD]	508.3 ± 160.6	505.1 ± 168.4	508.9 ± 159.3	0.803
NT-proBNP (pg/mL), mean ± SD	2320.1 ± 6833.4	8653.8 ± 14184.4	1227.2 ± 3482.8	**<0.001**
C-reactive protein (mg/L) [mean ± SD]	75.9 ± 65.8	100.3 ± 74.2	71.8 ± 63.4	**<0.001**
PCT (ng/mL) [mean ± SD]	0.7 ± 3.7	2.0 ± 7.8	0.4 ± 1.9	**<0.001**
IL-6 (ng/L) [mean ± SD]	54.9 ± 194.9	84.9 ± 168.7	50.4 ± 198.2	0.054
In-hospital outcomes				
Length of hospitalization (days) [mean ± SD]	16.2 ± 13.2	19.2 ± 17.9	15.6 ± 12.2	**0.002**
Diagnosis of myocarditis [*n*, (%)]	1 (0.1)	1 (0.7)	0 (0.0)	0.142
Positive haemoculture [*n*, (%)]	142 (13.9)	45 (31)	97 (11.1)	**<0.001**
Need for mechanic ventilation [*n*, (%)]	123 (12.1)	35 (24.1)	88 (10.1)	**<0.001**
Need for ICU admission [*n*, (%)]	212 (20.8)	58 (40.0)	154 (17.6)	**<0.001**
In-hospital deaths [*n*, (%)]	122 (12.0)	54 (37.2)	68 (7.8)	**<0.001**

AF, atrial fibrillation; CAD, coronary artery disease; CKD, chronic kidney disease; COVID-19, coronavirus disease 2019; DBP, diastolic blood pressure; eGFR, estimated glomerular filtration rate; Hb, haemoglobin; HF, heart failure; hs-cTnI, high-sensitivity cardiac troponin I; ICU, intensive care unit; IL-6, interleukin 6; LDH, lactate dehydrogenase; NT-proBNP, N-terminal prohormone of brain natriuretic peptide; PCR, polymerase chain reaction; PCT, procalcitonin; SARS-CoV-2, severe acute respiratory syndrome coronavirus 2; SBP, systolic blood pressure; SD, standard deviation; T2DM, type 2 diabetes mellitus; WBC, white blood cell count. Bold values represent statistically significant *p* values.

^a^Or equivalent (e.g. Jcovden/Janssen vaccine; see text for more details).

**Figure 1:**
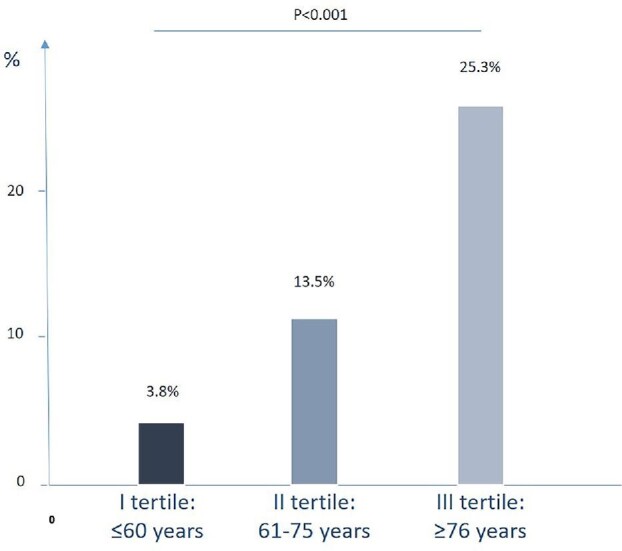
Prevalence of myocardial injury across age tertiles distribution.

Of importance, myocardial injury occurred more frequently in the III tertile (≥76 years) compared with other tertiles (25.3% in the III tertile vs. 13.5% in the II tertile vs. 3.8% in the I tertile; *P* < 0.001; *Figure 1*). Baseline characteristics and according to the presence or absence of myocardial injury in the I tertile (see Supplementary material online, Appendix *Table S2*), II tertile (see Supplementary material online, Appendix *Table S3*), and III tertile (see Supplementary material online, Appendix *Table S4*) are shown in the Supplementary Appendix online.

Moreover, the diagnosis of myocarditis occurred in only one (0.1% of the overall population) vaccinated patient in the I tertile. Of note, the clinical course of this myocarditis was mild and transient (no regional or global left ventricle dysfunction at echocardiography and cardiac magnetic resonance imaging), without adverse outcome (no need for mechanical ventilation or ICU admission). Finally, among patients identified with myocardial injury, 43 (29.7%) patients had both elevated troponin levels and ischaemic ECG abnormalities (13.8% in the III tertile vs. 10.3% in the II tertile vs. 5.5% in the I tertile; *P* < 0.055). Moreover, in patients with both elevated troponin levels and ischaemic ECG changes there were no differences between not vaccinated patients and vaccinated patients [27 (18.6%), 16 (11.0%); *P* = 0.44].

### Predictors of myocardial injury in the overall population

At multivariate logistic regression analysis, advanced age [OR: 1.053, CI 95% (1.036; 1.071), *P* < 0.001], CKD [OR: 2.082, 95% CI: (1.223; 3.543), *P* = 0.007], and hypertension [OR: 1.646, 95% CI: (1.112; 2.436), *P* = 0.013] were independent predictors for the occurrence of myocardial injury, while previous vaccination against COVID-19 was not significant [OR: 0.824, 95% CI: (0.556; 1.222), *P* = 0.336] (*Table 2*).

**Table 2:** Predictors of myocardial injury in the overall population by univariate and multivariate logistic regression analysis

	Univariate analysis		Multivariable analysis	
	OR (95% CI)	*P*	OR (95% CI)	*P*
Age	1.066 (1.050; 1.082)	**<0.001**	1.053 (1.036; 1.071)	**<0.001**
CKD	3.417 (2.119; 5.508)	**<0.001**	2.082 (1.223; 3.543)	**0.007**
T2DM	1.746 (1.150; 2.653)	**0.009**	0.999 (0.631; 1.582)	0.996
Hypertension	2.315 (1.603; 3.343)	**<0.001**	1.646 (1.112; 2.436)	**0.013**
History of HF	3.355 (1.923; 5.854)	**<0.001**	1.367 (0.712; 2.628)	0.348
Chronic obstructive pulmonary disease	1.953 (1.243; 3.067)	**0.004**	1.204 (0.731; 1.982)	0.466
Paroxysmal/persistent AF	3.265 (2.110; 5.052)	**<0.001**	1.470 (0.900; 2.402)	0.124
History of CAD	2.629 (1.722; 4.014)	**<0.001**	1.503 (0.939; 2.408)	0.090
Vaccination against COVID-19 (≥2 doses^a^)	1.455 (1.017; 2.082)	**0.040**	0.824 (0.556; 1.222)	0.336

AF, atrial fibrillation; CAD, coronary artery disease; CI, confidence interval; CKD, chronic kidney disease; HF, heart failure; OR, odds ratio; T2DM, type 2 diabetes mellitus. Bold values represent statistically significant *p* values.

^a^Or equivalent (e.g. Jcovden/Janssen vaccine; see text for more details).

### Predictors of myocardial injury according to age tertile distribution

In the I tertile (≤60 years), at multivariate logistic regression analysis, CKD (OR: 6.942, 95% CI: 1.310; 36.790, *P* = 0.023) and vaccination against COVID-19 (OR: 4.438, 95% CI: 1.284; 15.344, *P* = 0.019) were independent positive predictors for the occurrence of myocardial injury.

In the II tertile (61–75 years), at multivariate logistic regression analysis, CKD (OR: 3.115, 95% CI: 1.339; 7.246, *P* = 0.008), hypertension (OR: 2.095, 95% CI: 1.045; 4.201, *P* = 0.037), and COPD (OR: 2.369, 95% CI: 1.040; 5.395, *P* = 0.040) were independent positive predictors of myocardial injury, while vaccination status was not significant.

In the III tertile (≥76 years), at multivariate logistic regression analysis, vaccination against COVID-19 had a protective effect for myocardial injury (OR: 0.567, 95% CI: 0.341; 0.945, *P* = 0.029), while a previous history of CAD (OR: 1.906, 95% CI: 1.072; 3.390, *P* = 0.028) was an independent positive predictor (*Table 3*).

**Table 3:** Predictors of myocardial injury according to age tertiles distribution by univariate and multivariate logistic regression analysis

	Univariate analysis		Multivariable analysis	
	OR (95% CI)	*P*	OR (95% CI)	*P*
I tertile: ≤60 years				
CKD	16.000 (3.492; 73.312)	**<0.001**	6.942 (1.310–36.790)	**0.023**
Vaccination against COVID-19 (≥2 doses^a^)	6.440 (2.078; 19.961)	**0.001**	4.438 (1.284–15.344)	**0.019**
II tertile: 61–75 years				
CKD	3.273 (1.442; 7.430)	**0.005**	3.115 (1.339; 7.246)	**0.008**
Hypertension	2.404 (1.216; 4.751)	**0.012**	2.095 (1.045; 4.201)	**0.037**
Chronic obstructive pulmonary disease	2.444 (1.103; 5.418)	**0.028**	2.369 (1.040; 5.395)	**0.040**
Vaccination against COVID-19 (≥2 doses^a^)	1.248 (0.662; 2.351)	0.493	—	—
III tertile: ≥76 years				
History of HF	2.344 (1.148; 4.786)	**0.019**	1.716 (0.803; 3.667)	0.163
Paroxysmal/persistent AF	1.852 (1.087; 3.153)	**0.023**	1.682 (0.966; 2.928)	0.066
History of CAD	2.020 (1.167; 3.494)	**0.012**	1.906 (1.072; 3.390)	**0.028**
Vaccination against COVID-19 (≥2 doses^a^)	0.605 (0.368; 0.993)	**0.047**	0.567 (0.341; 0.945)	**0.029**

AF, atrial fibrillation; CAD, coronary artery disease; CI, confidence interval; CKD, chronic kidney disease; HF, heart failure; OR, odds ratio. Bold values represent statistically significant *p* values.

^a^Or equivalent (e.g. Jcovden/Janssen vaccine; see text for more details).

Finally, the interaction between age and COVID-19 vaccination status in relation to myocardial injury was statistically significant (*P* for interaction = 0.001).

### Sensitivity analysis for predictors of myocardial injury in the overall population and according to age tertile distribution

In the sensitivity analysis performed considering as vaccinated all patients who received one dose of COVID-19 vaccines, at multivariate logistic regression analysis, advanced age [OR: 1.052, 95% CI: (1.035; 1.070), *P* < 0.001], CKD [OR: 2.055, 95% CI: (1.211; 3.485), *P* = 0.008], and hypertension [OR: 1.644, 95% CI: (1.110; 2.628), *P* = 0.013] remained independent predictors for the occurrence of myocardial injury and vaccination against COVID-19 (≥1 dose) remained not significant [OR: 0.939, 95% CI: (0.635; 1.389), *P* = 0.754] in the overall population (see Supplementary material online, Appendix *Table S5*).

The same sensitivity analysis was also performed in each tertile according to age distribution (see Supplementary material online, Appendix *Table S6*). In the I tertile (≤60 years), at multivariate logistic regression analysis, CKD (OR: 6.836, 95% CI: 1.347; 34.694, *P* = 0.020) and vaccination against COVID-19 (≥1 dose) (OR: 6.635, 95% CI: 1.876; 23.473, *P* = 0.003) remained independent positive predictors for the occurrence of myocardial injury. In the II tertile (61–75 years), at multivariate logistic regression analysis, CKD (OR: 3.115, 95% CI: 1.339; 7.246, *P* = 0.008), hypertension (OR: 2.095, 95% CI: 1.045; 4.201, *P* = 0.037), and COPD (OR: 2.369, 95% CI: 1.040; 5.395, *P* = 0.040) remained independent positive predictors of myocardial injury and vaccination status remained not significant. Of note, in the III tertile (≥76 years), at multivariate logistic regression analysis, a previous history of CAD (OR: 1.790, 95% CI: 1.017; 3.150, *P* = 0.044) remained an independent positive predictor of myocardial injury, while vaccination against COVID-19 (≥1 dose) was not significant.

## Discussion

This study represents, to the best of our knowledge, the first study evaluating the incidence and predictors of myocardial injury after the introduction of COVID-19 vaccines and, in particular, the first study evaluating the role of vaccines in modifying the risk of myocardial injury associated with SARS-CoV-2 infection.

**Figure 2:**
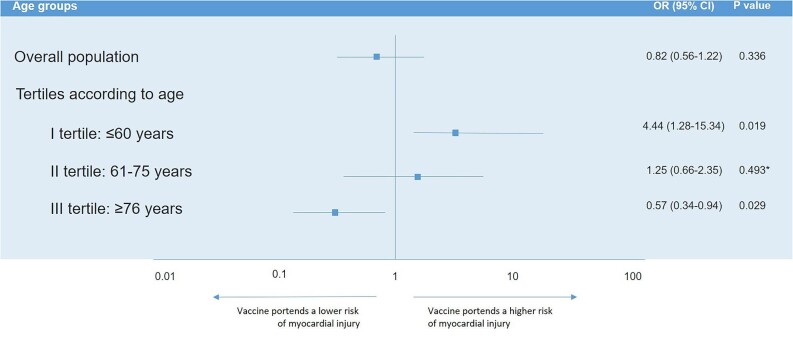
Adjusted risk of myocardial injury according to vaccination status in the overall population and across age tertiles distribution. *OR from univariate analysis. CI, confidence interval; OR, odds ratio.

The main results of our study can be summarized as follows: (i) the incidence of myocardial injury in patients hospitalized for COVID-19 is not negligible (14.2%) and varies according to age, being more prevalent in elder (25.3% among patients ≥76 years) than in younger patients (3.8% among patients ≤60 years); (ii) patients with myocardial injury were older, with a higher prevalence of pre-existing CV conditions, renal impairment, COPD, and T2DM, and had a more severe COVID-19 presentation as well as a worse in-hospital outcome; (iii) advanced age, CKD, and hypertension were the only independent predictors of myocardial injury in the overall population, whereas the role of vaccines in modifying the risk of myocardial injury was not significant; (iv) however, the effect of vaccines on the risk of myocardial injury varied according to age, being protective in the elderly (≥76 years) and, in contrast, possibly favouring its occurrence among younger patients (≤60 years) (*Figure 2*); and (v) the administration of a single dose of vaccine still favoured the occurrence of myocardial injury among patients ≤60 years but, of note, was not protective against its occurrence in the elderly (≥76 years).

The incidence of myocardial injury in our population was lower compared with previous studies conducted in the pre-vaccine era (ranging from 12% to 36%).^6–11^ However, different explanations may justify this finding, such as the variations in definition, population studied, timing of sampling and troponin assays/thresholds, the improvements in the management of COVID-19 patients, the development of different variants of SARS-CoV-2 (i.e. delta and omicron), and, more likely, the introduction of COVID-19 vaccines.^14,18^ Prior studies have highlighted the prognostic importance of myocardial injury in COVID-19 survivors, especially regarding post-discharge mortality.^19–21^ Consistent with these findings, we previously demonstrated that myocardial injury during hospitalization was associated with poorer outcomes, including an increased risk of mortality and a spectrum of CV complications after discharge, such as major CV and cerebrovascular events, arrhythmias, and inflammatory heart diseases (IHDs).^5^ Optimal management strategies for patients with COVID-19 who experience myocardial injury have yet to be definitively established. Nonetheless, treatment should be individualized, considering each patient's specific clinical circumstances. The pre-admission use of calcium channel blockers has not demonstrated a significant effect on all-cause mortality or illness severity in this patient group.^22^ For those presenting with symptoms of acute coronary syndrome, prompt and appropriate interventions, including coronary angiography and percutaneous coronary intervention, remain imperative. Given the increased risk of thrombo-embolic complications associated with severe COVID-19, prophylactic anticoagulation, particularly with heparin, is advised. The current body of evidence does not justify discontinuing angiotensin-converting enzyme inhibitors or angiotensin receptor blockers in the treatment of COVID-19 patients, although the impact of these agents continues to be the subject of ongoing research.^23^

We found that the incidence of myocardial injury was not equally distributed among the population as it varied greatly according to age, being more frequent in elder than in young patients. Moreover, in line with previous studies,^6–11,24,25^ we found that patients with myocardial injury were older, with a higher prevalence of comorbidities, a more severe COVID-19 presentation reflected by higher inflammatory and thrombotic markers, and a worse in-hospital outcome characterized by a higher need of mechanic ventilation, admission to ICU, and in-hospital deaths. Indeed, the risk for myocardial injury associated with acute infection (viral or bacterial) could be related to the increased inflammatory, prothrombotic, and procoagulant state, and might be promoted by advanced age and the presence of chronic CV conditions and/or an impaired renal function.^26^ Similarly, the occurrence of both non-ischaemic myocardial injury (i.e. myocarditis, stress cardiomyopathy, and acute HF) and type 1 and type 2 myocardial infarction might be fostered by COVID-19 infection in the elderly patients with chronic conditions and comorbidities.^27^ Accordingly, we identified advanced age, CKD, and hypertension as independent positive predictors of myocardial injury, while, surprisingly, COVID-19 vaccines had no effects in the overall population. However, by categorizing the overall population into three tertiles according to age distribution, we demonstrated for the first time that COVID-19 vaccines were protective against myocardial injury in the elderly population (≥76 years) reducing by approximately a half the risk of its occurrence, while, in sharp contrast, vaccines were associated with the occurrence of myocardial injury among patients ≤60 years, although it was a rare event in this group. Therefore, given that the former is the population at higher risk of myocardial injury associated with SARS-CoV-2 infection, the introduction of vaccines could explain the lower incidence of myocardial injury in our population, despite the increased risk in the younger. In addition, our results highlight the critical role of COVID-19 vaccination campaign in contrasting the harmful effects of SARS-CoV-2 infection, especially in the elderly. Furthermore, we also demonstrated that the protective effects of vaccines against myocardial injury in the elderly are lost when considering as vaccinated patients with one dose only of vaccine, thus supporting the importance of performing a full vaccination cycle in order to benefit from the positive effects of COVID-19 vaccines on the CV system.

Finally, the observation that COVID-19 vaccines are associated to an increased risk of myocardial injury among younger patients (≤60 years) might appear surprising and alarming. However, it must be noted that the incidence of myocardial injury in this group (3.8%) was a rather rare event. Furthermore, COVID-19 vaccines have already been associated with an increased, although low, risk of myocardial injury due to immune-mediated myocarditis, particularly in young individuals after the second dose of mRNA-based vaccines, showing, however, a benefit–risk profile favourable for COVID-19 vaccination and thus supporting the administration of vaccines.^18,28–32^ Likewise, the overall incidence of myocarditis in our population was extremely low (0.1%) without any effect on the clinical outcome. The potential interplay between pre-existing myocardial disease and myocardial injury due to COVID-19 is still unclear. Indeed, a recent study suggested that the clinical presentation and outcomes of vaccine-associated myocarditis may not significantly differ between patients with and with no previous diagnosis of myocarditis.^33^

Taken together, our findings allow to speculate that in the young the occurrence of myocardial injury could be related to different mechanisms compared with the elderly. Indeed, in the elderly the occurrence of myocardial injury might be mainly due to pre-existing CV disease or the development of *de novo* ischaemic or non-ischaemic mechanisms of myocardial injury fostered by the acute viral infection. In contrast, in the young, myocardial injury might be mainly related to the trigger of autoimmune mechanisms associated with SARS-CoV-2 infection that could be favoured by a previous immunization with COVID-19 vaccines. Accordingly, we found that young patients who experienced myocardial injury had a similar prevalence of comorbidities compared with those who did not but, of interest, had higher levels of circulating interleukin 6 (IL-6). Notably, higher levels of IL-6 have been associated both with a more intense immune activation during COVID-19 and with autoimmune and chronic inflammatory disease.^34–36^ However, our study remains hypothesis-generating and further larger studies are needed to confirm these results as well as to clarify whether autoimmune responses may explain these findings in young patients.

### Study limitations

Some limitations of our study should be acknowledged. First, this is a single-centre study. Second, despite being a prospectively enrolling real-time registry including all admitted COVID-19 patients, the observational retrospective nature of the analysis represents a limitation of this study. Third, there are limitations due to the use of EMR and NLP algorithms for data collection in such a large sample size not explicitly verified by manual chart review. Similarly, due to the exigencies posed by the COVID-19 pandemic, echocardiographies were not uniformly performed and/or reported across all patients, particularly in intensive care settings, resulting in a substantial proportion of missing data in this regard. Consequently, our study could not explore in detail the clinical meaning of the detected myocardial injury. Future studies are warranted to address these limitations and explore in detail the different types of myocardial injury. Fourth, cardiac troponin assessment was not routinely performed in COVID-19 patients, and this could have led to selection bias. Fifth, the real incidence of subclinical myocarditis and/or immune-mediated IHD is likely to be underestimated in our population, as many patients with myocardial injury not clinically significant did not probably undergo a comprehensive diagnostic work-up for the detection of myocarditis or IHD (e.g. cardiac magnetic resonance imaging, myocardial biopsy) also for epidemiological reasons. Finally, due to the variability in the timing of multiple troponin collections among patients, our study was limited in its ability to conduct a detailed analysis of myocardial injury patterns based on biomarker trends. Particularly, our cohort size, if segmented into different types of myocardial injury, would be too small to yield robust results. Future research, with more comprehensive data collection and larger cohorts, possibly applying machine learning techniques, is warranted to effectively distinguish between different myocardial injury patterns and provide deeper insights into their prognostic implications in COVID-19 patients.

## Conclusion

Myocardial injury is a relatively frequent complication associated with SARS-CoV-2 infection, and its incidence is higher in elderly patients. Of importance, we demonstrate for the first time that the effect of vaccine on the risk of myocardial injury may vary across different age strata. In particular, in patients ≥76 years COVID-19 vaccines seem to be protective for the occurrence of myocardial injury, while in patients ≤60 years the occurrence of myocardial injury associated with SARS-CoV-2 infection, despite being a rare event, might be favoured by previous COVID-19 vaccination. Further studies are needed to clarify the mechanisms responsible for the interaction between age and vaccination in determining COVID-19 infection–associated myocardial injury.

## Supplementary Material

qcae016_Supplemental_File

## Data availability

All data and methods supporting the findings of this study are available from the corresponding author upon reasonable request.
