# Supplementary material for: Vaccines and myocardial injury in patients hospitalized for COVID-19 infection: the CardioCOVID-Gemelli study
Source: Eur Heart J Qual Care Clin Outcomes. 2024 Feb 27;11(1):59–67. doi: 10.1093/ehjqcco/qcae016 (PMC11736151; doi:10.1093/ehjqcco/qcae016)
Supplement: qcae016_Supplemental_File [file qcae016_supplemental_file.docx]

**Online Appendix**

**Vaccines and Myocardial Injury in Patients Hospitalized for COVID-19 Infection: the CardioCOVID-Gemelli Study**

Rocco Antonio Montone^1^, MD, PhD; Riccardo Rinaldi^2^, MD; Carlotta Masciocchi^3^, PhD; Livia Lilli^3^, PhD; Andrea Damiani^4^, MD; Giulia La Vecchia^2^, MD; Giulia Iannaccone^2^, MD; Mattia Basile^2^, MD; Carmine Salzillo^2^, MD; Andrea Caffè^2^, MD; Alice Bonanni^1^, PhD; Gennaro De Pascale^5,6^, MD; Domenico Luca Grieco^5^, MD; Eloisa Sofia Tanzarella^5^, MD; Danilo Buonsenso^7^, MD, PhD; Rita Murri^4,8^, MD; Massimo Fantoni^4,8^, MD; Giovanna Liuzzo^1,2^, MD, PhD; Tommaso Sanna^1,2^, MD, PhD; Luca Richeldi^9^, MD, PhD; Maurizio Sanguinetti^10^, MD, PhD; Massimo Massetti^1,2^, MD; Carlo Trani^1,2^, MD; Yamume Tshomba^1,2^, MD, PhD; Antonio Gasbarrini^11^, MD, PhD; Vincenzo Valentini^12^, MD, PhD; Massimo Antonelli^5,6^, MD, PhD; Filippo Crea^1,2^, MD, PhD; on behalf of the Gemelli against COVID Group.

*^1^ Department of Cardiovascular Sciences, Fondazione Policlinico Universitario A. Gemelli IRCCS, Rome, Italy*

*^2^ Department of Cardiovascular and Pulmonary Sciences, Catholic University of the Sacred Heart*

*Rome, Italy*

*^3^Fondazione Policlinico Universitario A. Gemelli IRCCS, Roma, Italy.*

*^4^Dipartimento di Scienze di Laboratorio e Infettivologiche, Fondazione Policlinico Universitario A. Gemelli IRCCS, Roma, Italy*

*^5^ Department of Emergency, Intensive Care Medicine and Anaesthesia, Fondazione Policlinico Universitario A. Gemelli IRCCS; Rome, Italy*

*^6^ Istituto di Anestesiologia e Rianimazione, Università Cattolica del Sacro Cuore Rome, Italy*

*^7^ Department of Women's health, child health and public health sciences, Fondazione Policlinico Universitario A. Gemelli IRCCS, Rome, Italy*

*^8^Clinic of Infectious Diseases, Catholic University of the Sacred Heart, 00168 Rome, Italy*

*^9^Division of Pulmonary Medicine, Fondazione Policlinico Universitario Agostino Gemelli IRCCS, Università Cattolica del Sacro Cuore, Largo Agostino Gemelli 8, 00168, Rome, Italy*

*^10^Department of Basic Biotechnological Sciences, Intensive and Perioperative Clinics, Catholic University of the Sacred Heart, Rome, Italy*

*^11^Department of Medical and Surgical Sciences, Fondazione Policlinico Universitario A. Gemelli IRCCS, Rome, Italy; Department of Translational Medicine and Surgery, Catholic University of the Sacred Heart, Rome, Italy*

*^12^Department of Diagnostic Imaging, Radiotherapy, Oncology and Hematology, Fondazione Policlinico Universitario A. Gemelli IRCCS, Rome, Italy; Department of Radiological and Hematological Sciences, Catholic University of the Sacred Heart, Rome, Italy.*

***Corresponding author:***

**Prof. Filippo Crea, MD, FESC**

Department of Cardiovascular and Pulmonary Sciences,

Catholic University of the Sacred Heart

Largo F. Vito, 1 - 00168 Rome, Italy

Tel. +39-06-30154187

Fax +39-06-3055535

Email: filippo.crea@unicatt.it

**Legend:**

- **Appendix Main text**
- **Appendix Tables**
- **Appendix Figures**
- **Appendix Figure Legend**

***Appendix Main Text***

*Data collection*

Pre-existing conditions collected were chronic kidney disease (CKD) (defined as an estimated glomerular filtration rate < 60 ml/min per 1.73 m^2^), type 2 diabetes mellitus (T2DM), hypertension, history of heart failure (HF), chronic obstructive pulmonary disease (COPD), asthma, paroxysmal/persistent atrial fibrillation (AF), history of coronary artery disease (CAD), and malignancy. Vital signs included diastolic blood pressure (DBP) and systolic blood pressure (SBP) at the time of admission. Laboratory parameters included hematologic variables (haemoglobin and white blood cells), creatinine, total protein, antithrombin, high sensitivity cardiac troponin I (hs-cTnI), lactate dehydrogenase (LDH), D-dimer, fibrinogen, N-terminal Prohormone of Brain Natriuretic Peptide (NT-proBNP), C-reactive protein (CRP), procalcitonin (PCT) and interleukin 6 (IL-6) at the time of admission. For hs-cTnI, if multiple measurements were available within 30 days from admission, the patient’s first measurement was used and the peak value during the hospitalization was also collected. Data regarding patient’s vaccination status included the number of doses administered, the type of vaccine and the date of the last dose administered before the index hospitalization. Finally, data regarding patient’s clinical outcomes during the index hospitalization included the length of hospitalization, the need for mechanic ventilation, the need for admission in intensive care unit (ICU) and in-hospital deaths.

*Handling of missing data*

For categorical variables, no missing data were present due to the binary output capability of our Natural Language Processing (NLP) algorithms (indicating the presence or absence of a factor).

For continuous variables, missing data were recorded for several variables, which are listed below along with the approximate percentage of missingness:

- Diastolic Blood Pressure at admission (approximately 10% missing)

- Systolic Blood Pressure at admission (approximately 10% missing)

- Total protein (approximately 1% missing)

- Antithrombin (approximately 8% missing)

- Procalcitonin (approximately 23% missing)

- Interleukin 6 (approximately 19% missing)

- C-Reactive Protein (approximately 1% missing)

- N-terminal Prohormone of Brain Natriuretic Peptide (approximately 8% missing)

- D-dimer (approximately 2% missing).

The approach taken for handling missing data was to exclude them from the analysis. This decision was based on the proportion of missing data and the potential impact on the study's outcomes. Indeed, given the relatively low percentage of missing data for most variables, excluding these values was deemed unlikely to significantly affect the overall analysis.

***Appendix Tables***

**Appendix Table 1.** Baseline characteristics of the overall population and according to the time waves of COVID-19 infection.

| **Characteristics** | **Overall**  **Population**  (n= 1019) | | **Third wave**  **(From March 2021**  **to May 2021)**  (n= 310) | | **Post-Third wave**  **(From June 2021**  **to October 2021)**  (n= 286) | | **Fourth wave**  **(From November 2021**  **to March 2021)**  (n= 423) | **p value** |
| --- | --- | --- | --- | --- | --- | --- | --- | --- |
| ***Clinical characteristics*** |  |  | |  | |  | |  |
| Age [mean ± SD] | 67.7 ± 14.8 | 66.9 ± 14.7 | | 67.3 ± 15.6 | | 68.6 ± 14.3 | | 0.260 |
| Male sex [n, (%)] | 620 (60.8) | 185 (59.7) | | 169 (59.1) | | 266 (62.9) | | 0.526 |
| CKD (eGFR < 60 ml/min per 1.73 m^2^) [n, (%)] | 92 (9.0) | 20 (6.5) | | 22 (7.7) | | 50 (11.8) | | **0.028** |
| History of cancer [n, (%)] | 148 (14.5) | 39 (12.6) | | 35 (12.2) | | 74 (17.5) | | 0.076 |
| T2DM [n, (%)] | 175 (17.2) | 51 (16.5) | | 48 (16.8) | | 76 (18.0) | | 0.847 |
| Hypertension [n, (%)] | 489 (48.0) | 150 (48.4) | | 136 (47.6) | | 203 (48.0) | | 0.979 |
| History of HF [n, (%)] | 63 (6.2) | 20 (6.5) | | 12 (4.2) | | 31 (7.3) | | 0.230 |
| COPD [n, (%)] | 133 (13.1) | 32 (10.3) | | 37 (12.9) | | 64 (15.1) | | 0.161 |
| Asthma [n, (%)] | 40 (3.9) | 10 (3.2) | | 11 (3.8) | | 19 (4.5) | | 0.682 |
| Paroxysmal/persistent AF [n, (%)] | 120 (11.8) | 34 (11.0) | | 31 (10.8) | | 55 (13.0) | | 0.592 |
| History of CAD [n, (%)] | 142 (13.9) | 43 (13.9) | | 42 (14.7) | | 57 (13.5) | | 0.900 |
| DBP at admission (mmHg) [mean ± SD] | 79.0 ± 12.4 | 79.2 ± 12.4 | | 78.4 ± 12.1 | | 79.3 ± 12.6 | | 0.666 |
| SBP at admission (mmHg) [mean ± SD] | 132.6 ± 20.6 | 133.2 ± 20.6 | | 129.9 ± 20.2 | | 133.9 ± 20.6 | | 0.052 |
| ***Vaccination status*** |  |  | |  | |  | |  |
| Vaccinated (≥2 doses*) against COVID-19 [n, (%)] | 352 (34.5) | 43 (13.9) | | 118 (41.3) | | 191 (45.2) | | **<0.001** |
| Total COVID-19 vaccine doses administered [n, (%)] |  |  | |  | |  | | **<0.001** |
| Not vaccinated [n, (%)] | 601 (59.0) | 230 (74.0) | | 155 (54.2) | | 216 (51.1) | |  |
| 1 dose [n, (%)] | 66 (6.5) | 37 (11.9) | | 13 (4.5) | | 16 (3.8) | |  |
| 2 doses* [n, (%)] | 286 (28.1) | 37 (11.9) | | 113 (39.5) | | 136 (32.2) | |  |
| 3 doses [n, (%)] | 66 (6.5) | 6 (1.9) | | 5 (1.7) | | 55 (13.0) | |  |
| Type of COVID-19 vaccine [n, (%)] |  |  | |  | |  | | **<0.001** |
| Pfizer/BioNTech Comirnaty [n, (%)] | 184 (18.1) | 48 (15.4) | | 59 (20.6) | | 104 (24.6) | |  |
| Vaxzevria/AstraZeneca [n, (%)] | 27 (2.6) | 3 (1.0) | | 7 (2.4) | | 18 (4.3) | |  |
| Spikevax/Moderna [n, (%)] | 11 (1.1) | 1 (0.3) | | 2 (0.7) | | 9 (2.1) | |  |
| Jcovden/Janssen [n, (%)] | 5 (0.5) | 0 (0.0) | | 6 (2.1) | | 3 (0.7) | |  |
| Missing data [n, (%)] | 125 (12.3) | 13 (4.2) | | 48 (16.8) | | 64 (15.1) | |  |
| Time from last dose of vaccine to troponin assessment (days) [mean ± standard deviation] | 122.9 ± 84.7 | 63.5 ± 81.5 | | 119.2 ± 67.5 | | 147.0 ± 83.7 | | **<0.001** |
| Time from last dose of vaccine to positive PCR test for SARS-CoV-2 (days) [mean ± standard deviation] | 121.0 ± 84.5 | 61.6 ± 80.8 | | 118.1 ± 67.2 | | 145.1 ± 83.7 | | **<0.001** |
| ***Laboratory data*** |  |  | |  | |  | |  |
| Hb (g/dL) [mean ± SD] | 13.5 ± 2.1 | 13.6 ± 2.1 | | 13.6 ± 1.9 | | 13.5 ± 2.2 | | 0.526 |
| WBC (x10^9^/L) [mean ± SD] | 8.0 ± 6.3 | 8.2 ± 7.3 | | 7.5 ± 4.0 | | 8.2 ± 6.8 | | 0.303 |
| Serum creatinine on admission (mg/dL) [mean ± SD] | 1.2 ± 1.3 | 1.1 ± 0.8 | | 1.1 ± 0.7 | | 1.3 ± 1.7 | | **0.024** |
| Total protein (g/L) [mean ± SD] | 66.2 ± 6.6 | 66.0 ± 6.5 | | 67.2 ± 6.0 | | 65.7 ± 7.1 | | **0.014** |
| Antithrombin (%) [mean ± SD] | 101.4 ± 17.5 | 99.9 ± 16.5 | | 104.0 ± 18.2 | | 100.6 ± 17.5 | | **0.013** |
| hs-cTnI at admission (ng/L) [mean ± SD] | 263.5 ± 3398.5 | 451.8 ± 4920.9 | | 127.3 ± 1066.1 | | 217.5 ± 3053.2 | | 0.475 |
| hs-cTnI peak during index hospitalization (ng/L) [mean ± SD] | 459.5 ± 4456.1 | 930.8 ± 7003.1 | | 162.2 ± 119.5 | | 315.2 ± 3301.5 | | 0.075 |
| LDH (UI/L) [mean ± SD] | 406.6 ± 297.0 | 386.8 ± 193.0 | | 404.5 ± 217.4 | | 422.5 ± 391.0 | | 0.273 |
| D-dimer (ng/mL) [mean ± SD] | 2238.9 ± 4920.8 | 2294.7 ± 5254.7 | | 2018.0 ± 4588.1 | | 2349.7 ± 4898.1 | | 0.664 |
| Fibrinogen (mg/dL) [mean ± SD] | 508.3 ± 160.6 | 523.2 ± 159.6 | | 556.3 ± 173.5 | | 464.9 ± 140.0 | | **<0.001** |
| NT-proBNP (pg/mL) [mean ± SD] | 2320.1 ± 6833.4 | 2339.8 ± 5231.3 | | 2284.8 ± 9487.8 | | 2330.3 ± 5566.6 | | 0.995 |
| CRP (mg/L) [mean ± SD] | 75.9 ± 65.8 | 72.3 ± 64.7 | | 83.1 ± 66.3 | | 73.7 ± 65.9 | | 0.090 |
| PCT (ng/mL) [mean ± SD] | 0.7 ± 3.7 | 0.4 ± 1.3 | | 0.6 ± 3.9 | | 1.0 ± 4.6 | | 0.134 |
| IL-6 (ng/L) [mean ± SD] | 54.9 ± 194.9 | 42.2 ± 70.6 | | 65.1 ± 319.2 | | 56.6 ± 134.2 | | 0.436 |
| ***In-hospital outcomes*** |  |  | |  | |  | |  |
| Length of hospitalization (days) [mean ± SD] | 16.2 ± 13.2 | 16.6 ± 14.8 | | 17.4 ± 14.3 | | 15.0 ± 10.9 | | **0.038** |
| Myocardial injury [n, (%)] | 145 (14.2) | 47 (15.2) | | 35 (12.2) | | 63 (14.9) | | 0.521 |
| Diagnosis of myocarditis [n, (%)] | 1 (0.1) | 0 (0.0) | | 1 (0.3) | | 0 (0.0) | | 0.277 |
| Need for mechanic ventilation [n, (%)] | 123 (12.1) | 52 (16.8) | | 33 (11.5) | | 38 (9.0) | | **0.006** |
| Need for ICU admission [n, (%)] | 212 (20.8) | 89 (28.7) | | 61 (21.3) | | 62 (14.7) | | **<0.001** |
| In-hospital deaths [n, (%)] | 122 (12.0) | 41 (13.2) | | 39 (13.6) | | 42 (9.9) | | 0.236 |
|  |  | |  | |  | |  |  |

**Legend:** SD: Standard Deviation; CKD: Chronic Kidney Disease; T2DM: Type 2 Diabetes Mellitus; eGFR: estimated Glomerular Filtration Rate; HF: Heart Failure; COPD: Chronic Obstructive Pulmonary Disease; AF: Atrial Fibrillation; CAD: Coronary Artery Disease; DBP: Diastolic Blood Pressure; SBP: Systolic Blood Pressure; COVID-19: Coronavirus Disease 2019; PCR: Polymerase Chain Reaction; SARS-CoV-2: Severe Acute Respiratory Syndrome Coronavirus 2; Hb: Haemoglobin; WBC: White Blood Count; hs-cTnI: high sensitivity cardiac troponin I; LDH: Lactate Dehydrogenase; NT-proBNP: N-terminal Prohormone of Brain Natriuretic Peptide; CRP: C Reactive Protein; PCT: Procalcitonin; IL-6: Interleukin 6; ICU: Intensive Care Unit.

*or equivalent (e.g.: Jcovden/Janssen vaccine; see text for more details).

**Appendix Table 2.** Baseline characteristics of the I tertile of population according to age (≤60 years) and according to the presence or absence of myocardial injury.

| **Characteristics** | | **Overall**  **population**  (n= 329) | | **Patients with**  **myocardial injury**  (n= 13) | | **Patients without**  **myocardial injury**  (n = 316) | | **p value** | |
| --- | --- | --- | --- | --- | --- | --- | --- | --- | --- |
| ***Clinical characteristics*** |  | |  | |  | |  | |  |
| Age [mean ± SD] | 50.5 ± 8.3 | | 48.5 ± 12.2 | | 50.6 ± 8.1 | | 0.384 | |  |
| Male sex [n, (%)] | 236 (71.7) | | 11 (84.6) | | 225 (71.2) | | 0.365 | |  |
| CKD (eGFR < 60 ml/min per 1.73 m^2^) [n, (%)] | 8 (2.4) | | 3 (23.1) | | 5 (1.6) | | **0.002** | |  |
| History of cancer [n, (%)] | 27 (8.2) | | 1 (7.7) | | 26 (8.2) | | 1.000 | |  |
| T2DM [n, (%)] | 20 (6.1) | | 1 (7.7) | | 19 (6.0) | | 0.564 | |  |
| Hypertension [n, (%)] | 96 (29.2) | | 4 (30.8) | | 92 (29.1) | | 1.000 | |  |
| History of HF [n, (%)] | 4 (1.2) | | 1 (7.7) | | 3 (0.9) | | 0.150 | |  |
| COPD [n, (%)] | 15 (4.6) | | 2 (15.4) | | 13 (4.1) | | 0.113 | |  |
| Asthma [n, (%)] | 18 (5.5) | | 1 (7.7) | | 17 (5.4) | | 0.526 | |  |
| Paroxysmal/persistent AF [n, (%)] | 8 (2.4) | | 1 (7.7) | | 7 (2.2) | | 0.278 | |  |
| History of CAD [n, (%)] | 14 (4.3) | | 1 (7.7) | | 13 (4.1) | | 0.438 | |  |
| DBP at admission (mmHg) [mean ± SD] | 81.5 ± 11.4 | | 79.9 ± 14.9 | | 81.5 ± 11.3 | | 0.738 | |  |
| SBP at admission (mmHg) [mean ± SD] | 130.6 ± 17.8 | | 126.9 ± 22.5 | | 130.7 ± 17.7 | | 0.605 | |  |
| Vaccinated (≥2 doses*) against COVID-19 [n, (%)] | 56 (17.0) | | 7 (53.8) | | 49 (15.6) | | **<0.001** | |  |
| Total COVID-19 vaccine doses administered [n, (%)] |  | |  | |  | | **<0.001** | |  |
| Not vaccinated [n, (%)] | 255 (77.5) | | 4 (30.8) | | 251 (79.4) | |  | |  |
| 1 dose [n, (%)] | 18 (5.5) | | 2 (15.4) | | 16 (5.1) | |  | |  |
| 2 doses* [n, (%)] | 44 (13.4) | | 4 (30.8) | | 40 (12.7) | |  | |  |
| 3 doses [n, (%)] | 12 (3.6) | | 3 (23.1) | | 9 (2.8) | |  | |  |
| ***Laboratory data*** |  | |  | |  | |  | |  |
| Hb (g/dL) [mean ± SD] | 14.5 ± 1.7 | | 14.0 ± 2.0 | | 14.5 ± 1.7 | | 0.390 | |  |
| WBC (x10^9^/L) [mean ± SD] | 7.5 ± 3.6 | | 11.6 ± 5.3 | | 7.3 ± 3.4 | | **<0.001** | |  |
| Serum creatinine on admission (mg/dL) [mean ± SD] | 0.9 ± 0.8 | | 1.2 ± 1.1 | | 0.9 ± 0.8 | | **0.044** | |  |
| Total protein (g/L) [mean ± standard deviation] | 68.2 ± 5.6 | | 63.8 ± 7.4 | | 68.3 ± 5.5 | | 0.060 | |  |
| Antithrombin (%) [mean ± SD] | 107.7 ± 17.0 | | 91.7 ± 21.4 | | 108.3 ± 16.7 | | **0.037** | |  |
| hs-cTnI at admission (ng/L) [mean ± SD] | 329.4 ± 4741.8 | | 8130.5 ± 23365.3 | | 8.5 ± 8.7 | | **<0.001** | |  |
| hs-cTnI peak during index hospitalization (ng/L) [mean ± SD] | 334.3 ± 4742.8 | | 8169.9 ± 22352.7 | | 11.9 ± 43.7 | | **<0.001** | |  |
| LDH (UI/L) [mean ± SD] | 420.5 ± 241.9 | | 424.7 ± 206.4 | | 420.3 ± 243.4 | | 0.945 | |  |
| D-dimer (ng/mL) [mean ± SD] | 1337.8 ± 3587.1 | | 1913.2 ± 1267.3 | | 1315.6 ± 3646.2 | | 0.173 | |  |
| Fibrinogen (mg/dL) [mean ± SD] | 516.1 ± 160.9 | | 488.0 ± 220.7 | | 517.3 ± 158.3 | | 0.830 | |  |
| NT-proBNP (pg/mL) [mean ± SD] | 449.7 ± 1621.6 | | 2779.3 ± 4439.0 | | 368.1 ± 1381.6 | | **<0.001** | |  |
| CRP (mg/dL) [mean ± SD] | 69.3 ± 61.2 | | 89.6 ± 66.7 | | 68.5 ± 60.9 | | 0.221 | |  |
| PCT (ng/mL) [mean ± SD] | 0.3 ± 0.9 | | 0.5 ± 0.6 | | 0.3 ± 0.9 | | 0.339 | |  |
| IL-6 (ng/mL) [mean ± SD] | 47.8 ± 119.5 | | 264.4 ± 467.3 | | 41.6 ± 86.5 | | **<0.001** | |  |
|  | |  | |  | |  | |  | |

**Legend to table:** SD: Standard Deviation; CKD: Chronic Kidney Disease; T2DM: Type 2 Diabetes Mellitus; eGFR: estimated Glomerular Filtration Rate; HF: Heart Failure; COPD: Chronic Obstructive Pulmonary Disease; AF: Atrial Fibrillation; CAD: Coronary Artery Disease; DBP: Diastolic Blood Pressure; SBP: Systolic Blood Pressure; COVID-19: Coronavirus Disease 2019; Hb: Haemoglobin; WBC: White Blood Count; hs-cTnI: high sensitivity cardiac troponin I; LDH: Lactate Dehydrogenase; NT-proBNP: N-terminal Prohormone of Brain Natriuretic Peptide; CRP: C Reactive Protein; PCT: Procalcitonin; IL-6: Interleukin 6; ICU: Intensive Care Unit.

*or equivalent (e.g.: Jcovden/Janssen vaccine; see text for more details).

**Appendix Table 3.** Baseline characteristics of the II tertile of population according to age (61-74 years) and according to the presence or absence of myocardial injury.

| **Characteristics** | | **Overall**  **population**  (n= 322) | | **Patients with**  **myocardial injury**  (n= 37) | | **Patients without**  **myocardial injury**  (n = 285) | | **p value** | |
| --- | --- | --- | --- | --- | --- | --- | --- | --- | --- |
| ***Clinical characteristics*** |  | |  | |  | |  | |  |
| Age [mean ± SD] | 67.7 ± 4.2 | | 68.1 ± 4.1 | | 67.6 ± 4.2 | | 0.530 | |  |
| Male sex [n, (%)] | 197 (61.2) | | 24 (64.9) | | 173 (60.7) | | 0.625 | |  |
| CKD (eGFR < 60 ml/min per 1.73 m^2^) [n, (%)] | 31 (9.6) | | 8 (21.6) | | 23 (8.1) | | **0.009** | |  |
| History of cancer [n, (%)] | 55 (17.1) | | 6 (16.2) | | 49 (17.2) | | 0.882 | |  |
| T2DM [n, (%)] | 64 (19.9) | | 9 (24.3) | | 55 (19.3) | | 0.471 | |  |
| Hypertension [n, (%)] | 162 (50.3) | | 24 (64.9) | | 138 (48.4) | | 0.060 | |  |
| History of HF [n, (%)] | 17 (5.3) | | 2 (5.4) | | 15 (5.3) | | 1.000 | |  |
| COPD [n, (%)] | 36 (11.2) | | 8 (21.6) | | 28 (9.8) | | **0.032** | |  |
| Asthma [n, (%)] | 10 (3.1) | | 0 (0.0) | | 10 (3.5) | | 0.612 | |  |
| Paroxysmal/persistent AF [n, (%)] | 16 (5.0) | | 2 (5.4) | | 14 (4.9) | | 0.704 | |  |
| History of CAD [n, (%)] | 45 (14.0) | | 5 (13.5) | | 40 (14.0) | | 1.000 | |  |
| DBP at admission (mmHg) [mean ± SD] | 78.9 ± 11.6 | | 81.0 ± 14.1 | | 78.6 ± 11.3 | | 0.294 | |  |
| SBP at admission (mmHg) [mean ± SD] | 132.3 ± 20.0 | | 133.0 ± 23.1 | | 132.2 ± 19.7 | | 0.861 | |  |
| Vaccinated (≥2 doses*) against COVID-19 [n, (%)] | 114 (35.4) | | 16 (43.2) | | 98 (34.4) | | 0.289 | |  |
| Total COVID-19 vaccine doses administered [n, (%)] |  | |  | |  | | 0.0540 | |  |
| Not vaccinated [n, (%)] | 188 (58.4) | | 18 (48.6) | | 170 (59.6) | |  | |  |
| 1 dose [n, (%)] | 20 (6.2) | | 3 (8.1) | | 17 (6.0) | |  | |  |
| 2 doses* [n, (%)] | 93 (28.9) | | 14 (37.8) | | 79 (27.7) | |  | |  |
| 3 doses [n, (%)] | 21 (6.5) | | 2 (5.4) | | 19 (6.7) | |  | |  |
| ***Laboratory data*** |  | |  | |  | |  | |  |
| Hb (g/dL) [mean ± SD] | 13.6 ± 2.0 | | 12.1 ± 2.4 | | 13.8 ± 1.9 | | **<0.001** | |  |
| WBC (x10^9^/L) [mean ± SD] | 8.4 ± 8.5 | | 10.1 ± 6.7 | | 8.2 ± 8.7 | | 0.126 | |  |
| Serum creatinine on admission (mg/dL) [mean ± SD] | 1.3 ± 1.5 | | 2.1 ± 3.1 | | 1.2 ± 1.1 | | **0.001** | |  |
| Total protein (g/L) [mean ± standard deviation] | 66.1 ± 6.9 | | 65.6 ± 7.9 | | 66.1 ± 6.8 | | 0.722 | |  |
| Antithrombin (%) [mean ± SD] | 102.5 ± 16.7 | | 98.9 ± 14.7 | | 103.0 ± 17.0 | | 0.140 | |  |
| hs-cTnI at admission (ng/L) [mean ± SD] | 101.6 ± 624.7 | | 779.7 ± 1716.1 | | 13.6 ± 12.6 | | **<0.001** | |  |
| hs-cTnI peak during index hospitalization (ng/L) [mean ± SD] | 221.7 ± 1519.0 | | 1674.7 ± 4224.9 | | 33.1 ± 185.4 | | **<0.001** | |  |
| LDH (UI/L) [mean ± SD] | 415.7 ± 393.2 | | 566.6 ± 952.4 | | 396.1 ± 237.6 | | **0.013** | |  |
| D-dimer (ng/mL) [mean ± SD] | 1966.7 ± 4131.2 | | 5350.2 ± 8852.8 | | 1524.4 ± 2757.3 | | **<0.001** | |  |
| Fibrinogen (mg/dL) [mean ± SD] | 515.5 ± 160.3 | | 542.3 ± 199.5 | | 512.0 ± 154.6 | | 0.281 | |  |
| NT-proBNP (pg/mL) [mean ± SD] | 1419.5 ± 4078.8 | | 4769.5 ± 8165.5 | | 997.6 ± 2993.7 | | **<0.001** | |  |
| CRP (mg/dL) [mean ± SD] | 79.1 ± 65.6 | | 104.8 ± 77.4 | | 75.8 ± 63.3 | | **0.011** | |  |
| PCT (ng/mL) [mean ± SD] | 0.9 ± 5.2 | | 4.5 ± 14.3 | | 0.3 ± 0.9 | | **<0.001** | |  |
| IL-6 (ng/mL) [mean ± SD] | 71.2 ± 315.3 | | 83.4 ± 170.3 | | 70.0 ± 326.6 | | 0.843 | |  |
|  | |  | |  | |  | |  | |

**Legend to table:** SD: Standard Deviation; CKD: Chronic Kidney Disease; T2DM: Type 2 Diabetes Mellitus; eGFR: estimated Glomerular Filtration Rate; HF: Heart Failure; COPD: Chronic Obstructive Pulmonary Disease; AF: Atrial Fibrillation; CAD: Coronary Artery Disease; DBP: Diastolic Blood Pressure; SBP: Systolic Blood Pressure; COVID-19: Coronavirus Disease 2019; Hb: Haemoglobin; WBC: White Blood Count; hs-cTnI: high sensitivity cardiac troponin I; LDH: Lactate Dehydrogenase; NT-proBNP: N-terminal Prohormone of Brain Natriuretic Peptide; CRP: C Reactive Protein; PCT: Procalcitonin; IL-6: Interleukin 6; ICU: Intensive Care Unit.

*or equivalent (e.g.: Jcovden/Janssen vaccine; see text for more details).

**Appendix Table 4.** Baseline characteristics of the III tertile of population according to age (≥76 years) and according to the presence or absence of myocardial injury.

| **Characteristics** | | **Overall**  **population**  (n= 368) | | **Patients with**  **myocardial injury**  (n= 95) | | **Patients without**  **myocardial injury**  (n = 273) | | **p value** | |
| --- | --- | --- | --- | --- | --- | --- | --- | --- | --- |
| ***Clinical characteristics*** |  | |  | |  | |  | |  |
| Age [mean ± SD] | 83.0 ± 5.6 | | 84.9 ± 6.3 | | 82.4 ± 5.1 | | **<0.001** | |  |
| Male sex [n, (%)] | 187 (50.8) | | 58 (61.1) | | 129 (47.3) | | **0.020** | |  |
| CKD (eGFR < 60 ml/min per 1.73 m^2^) [n, (%)] | 53 (14.4) | | 19 (20.0) | | 34 (12.5) | | 0.071 | |  |
| History of cancer [n, (%)] | 66 (17.9) | | 15 (15.8) | | 51 (18.7) | | 0.527 | |  |
| T2DM [n, (%)] | 91 (24.7) | | 26 (27.4) | | 65 (23.8) | | 0.489 | |  |
| Hypertension [n, (%)] | 230 (62.5) | | 66 (69.5) | | 164 (60.1) | | 0.103 | |  |
| History of HF [n, (%)] | 42 (11.4) | | 18 (18.9) | | 24 (8.8) | | **0.007** | |  |
| COPD [n, (%)] | 82 (22.3) | | 20 (21.1) | | 62 (22.7) | | 0.738 | |  |
| Asthma [n, (%)] | 12 (3.3) | | 4 (4.2) | | 8 (2.9) | | 0.545 | |  |
| Paroxysmal/persistent AF [n, (%)] | 96 (26.1) | | 34 (35.8) | | 62 (22.7) | | **0.012** | |  |
| History of CAD [n, (%)] | 82 (22.6) | | 32 /33.7) | | 51 (18.7) | | **0.003** | |  |
| DBP at admission (mmHg) [mean ± SD] | 76.9 ± 13.5 | | 74.4 ± 12.6 | | 77.7 ± 13.7 | | 0.064 | |  |
| SBP at admission (mmHg) [mean ± SD] | 134.6 ± 23.3 | | 129.5 ± 23.3 | | 136.3 ± 23.2 | | **0.026** | |  |
| Vaccinated (≥2 doses*) against COVID-19 [n, (%)] | 182 (49.5) | | 38 (40.0) | | 144 (52.7) | | **0.032** | |  |
| Total COVID-19 vaccine doses administered [n, (%)] |  | |  | |  | | 0.091 | |  |
| Not vaccinated [n, (%)] | 158 (42.9) | | 48 (50.5) | | 110 (40.3) | |  | |  |
| 1 dose [n, (%)] | 28 (7.6) | | 9 (9.5) | | 19 (7.0) | |  | |  |
| 2 doses* [n, (%)] | 153 (41.6) | | 29 (30.5) | | 124 (45.4) | |  | |  |
| 3 doses [n, (%)] | 29 (7.9) | | 9 (9.5) | | 20 (7.3) | |  | |  |
| ***Laboratory data*** |  | |  | |  | |  | |  |
| Hb (g/dL) [mean ± SD] | 12.7 ± 2.1 | | 12.7 ± 2.0 | | 12.6 ± 2.2 | | 0.473 | |  |
| WBC (x10^9^/L) [mean ± SD] | 8.2 ± 5.9 | | 10.3 ± 9.3 | | 7.5 ± 3.9 | | **<0.001** | |  |
| Serum creatinine on admission (mg/dL) [mean ± SD] | 1.3 ± 1.3 | | 1.5 ± 0.9 | | 1.2 ± 1.4 | | 0.066 | |  |
| Total protein (g/L) [mean ± standard deviation] | 64.6 ± 6.8 | | 64.0 ± 7.1 | | 64.8 ± 6.7 | | 0.342 | |  |
| Antithrombin (%) [mean ± SD] | 94.5 ± 16.1 | | 90.0 ± 15.7 | | 96.0 ± 16.0 | | **0.003** | |  |
| hs-cTnI at admission (ng/L) [mean ± SD] | 364.1 ± 3400.9 | | 1285.1 ± 6629.9 | | 19.3 ± 13.1 | | **0.002** | |  |
| hs-cTnI peak during index hospitalization (ng/L) [mean ± SD] | 779.4 ± 5726.9 | | 2953.9 ± 11027.5 | | 22.7 ± 25.6 | | **<0.001** | |  |
| LDH (UI/L) [mean ± SD] | 384.3 ± 235.3 | | 431.5 ± 252.3 | | 367.9 ± 227.3 | | **0.023** | |  |
| D-dimer (ng/mL) [mean ± SD] | 3306.6 ± 6254.5 | | 4940.4 ± 7967.1 | | 2753.9 ± 5465.6 | | **0.004** | |  |
| Fibrinogen (mg/dL) [mean ± SD] | 495.3 ± 160.4 | | 494.0 ± 147.3 | | 495.8 ± 165.1 | | 0.919 | |  |
| NT-proBNP (pg/mL) [mean ± SD] | 4468.1 ± 9852.1 | | 10732.1 ± 16053.6 | | 2286.3 ± 4834.2 | | **<0.001** | |  |
| CRP (mg/dL) [mean ± SD] | 79.1 ± 69.5 | | 100.2 ± 74.6 | | 71.7 ± 66.2 | | **0.001** | |  |
| PCT (ng/mL) [mean ± SD] | 0.8 ± 3.5 | | 1.3 ± 4.2 | | 0.6 ± 3.1 | | 0.183 | |  |
| IL-6 (ng/mL) [mean ± SD] | 47.0 ± 69.7 | | 69.2 ± 91.2 | | 39.4 ± 59.0 | | **0.001** | |  |
|  | |  | |  | |  | |  | |

**Legend to table:** SD: Standard Deviation; CKD: Chronic Kidney Disease; T2DM: Type 2 Diabetes Mellitus; eGFR: estimated Glomerular Filtration Rate; HF: Heart Failure; COPD: Chronic Obstructive Pulmonary Disease; AF: Atrial Fibrillation; CAD: Coronary Artery Disease; DBP: Diastolic Blood Pressure; SBP: Systolic Blood Pressure; COVID-19: Coronavirus Disease 2019; Hb: Haemoglobin; WBC: White Blood Count; hs-cTnI: high sensitivity cardiac troponin I; LDH: Lactate Dehydrogenase; NT-proBNP: N-terminal Prohormone of Brain Natriuretic Peptide; CRP: C Reactive Protein; PCT: Procalcitonin; IL-6: Interleukin 6; ICU: Intensive Care Unit.

*or equivalent (e.g.: Jcovden/Janssen vaccine; see text for more details).

**Appendix Table 5.** Predictors of myocardial injury in the overall population by univariate and multivariate logistic regression analysis.

|  | **Univariate analysis** | | **Multivariable analysis** | |
| --- | --- | --- | --- | --- |
|  | **OR (95% C.I.)** | **p** | **OR (95% C.I.)** | **p** |
| Age | 1.066 (1.050; 1.082) | **<0.001** | 1.052 (1.035; 1.070) | **<0.001** |
| CKD | 3.417 (2.119; 5.508) | **<0.001** | 2.055 (1.211; 3.485) | **0.008** |
| T2DM | 1.746 (1.150; 2.653) | **0.009** | 0.994 (0.628; 1.573) | 0.978 |
| Hypertension | 2.315 (1.603; 3.343) | **<0.001** | 1.644 (1.110; 2.434) | **0.013** |
| History of HF | 3.355 (1.923; 5.854) | **<0.001** | 1.372 (0.716; 2.628) | 0.341 |
| COPD | 1.953 (1.243; 3.067) | **0.004** | 1.185 (0.720; 1.950) | 0.504 |
| Paroxysmal/Persistent AF | 3.265 (2.110; 5.052) | **<0.001** | 1.465 (0.897; 2.390) | 0.127 |
| History of CAD | 2.629 (1.722; 4.014) | **<0.001** | 1.489 (0.929; 2.387) | 0.098 |
| Vaccination against COVID-19 (≥1 dose) | 1.659 (1.165; 2.361) | **0.005** | 0.939 (0.635; 1.389) | 0.754 |

**Legend**: OR: Odds Ratio; CI: Confidence Interval; CKD: Chronic Kidney Disease; T2DM: Type 2 Diabetes Mellitus; HF: Heart Failure; COPD: Chronic Obstructive Pulmonary Disease; AF: Atrial Fibrillation; CAD: Coronary Artery Disease.

**Appendix Table 6.** Sensitivity analysis of predictors of myocardial injury according to age tertiles by univariate and multivariate logistic regression analysis.

|  | **Univariate analysis** | | **Multivariable analysis** | |
| --- | --- | --- | --- | --- |
|  | **OR (95% C.I.)** | **p** | **OR (95% C.I.)** | **p** |
| **I tertile: ≤60 years** |  |  |  |  |
| CKD | 16.000 (3.492; 73.312) | **<0.001** | 6.836 (1.347; 34.694) | **0.020** |
| Vaccination against COVID-19 (≥1 dose) | 8.698 (2.599; 29.111) | **<0.001** | 6.635 (1.876; 23.473) | **0.003** |
| **II tertile: 61-75 years** |  |  |  |  |
| CKD | 3.273 (1.442; 7.430) | **0.005** | 3.115 (1.339; 7.246) | **0.008** |
| Hypertension | 2.404 (1.216; 4.751) | **0.012** | 2.095 (1.045; 4.201) | **0.037** |
| COPD | 2.444 (1.103; 5.418) | **0.028** | 2.369 (1.040; 5.395) | **0.040** |
| Vaccination against COVID-19 (≥1 dose) | 1.517 (0.813; 2.828) | 0.190 | - | **-** |
| **III tertile: ≥76 year** |  |  |  |  |
| History of HF | 2.344 (1.148; 4.786) | **0.019** | 1.777 (0.842; 3.748) | 0.131 |
| Paroxysmal/Persistent AF | 1.852 (1.087; 3.153) | **0.023** | 1.646 (0.951: 2.850) | 0.075 |
| History of CAD | 2.020 (1.167; 3.494) | **0.012** | 1.790 (1.017; 3.150) | **0.044** |
| Vaccination against COVID-19 (≥1 dose) | 0.630 (0.385; 1.030) | 0.066 | - | **-** |

**Legend**: OR: Odds Ratio; CI: Confidence Interval; CKD: Chronic Kidney Disease; COPD: Chronic Obstructive Pulmonary Disease; HF: Heart Failure; AF: Atrial Fibrillation; CAD: Coronary Artery Disease.

***Appendix Figures***

**Appendix Figure 1**

**
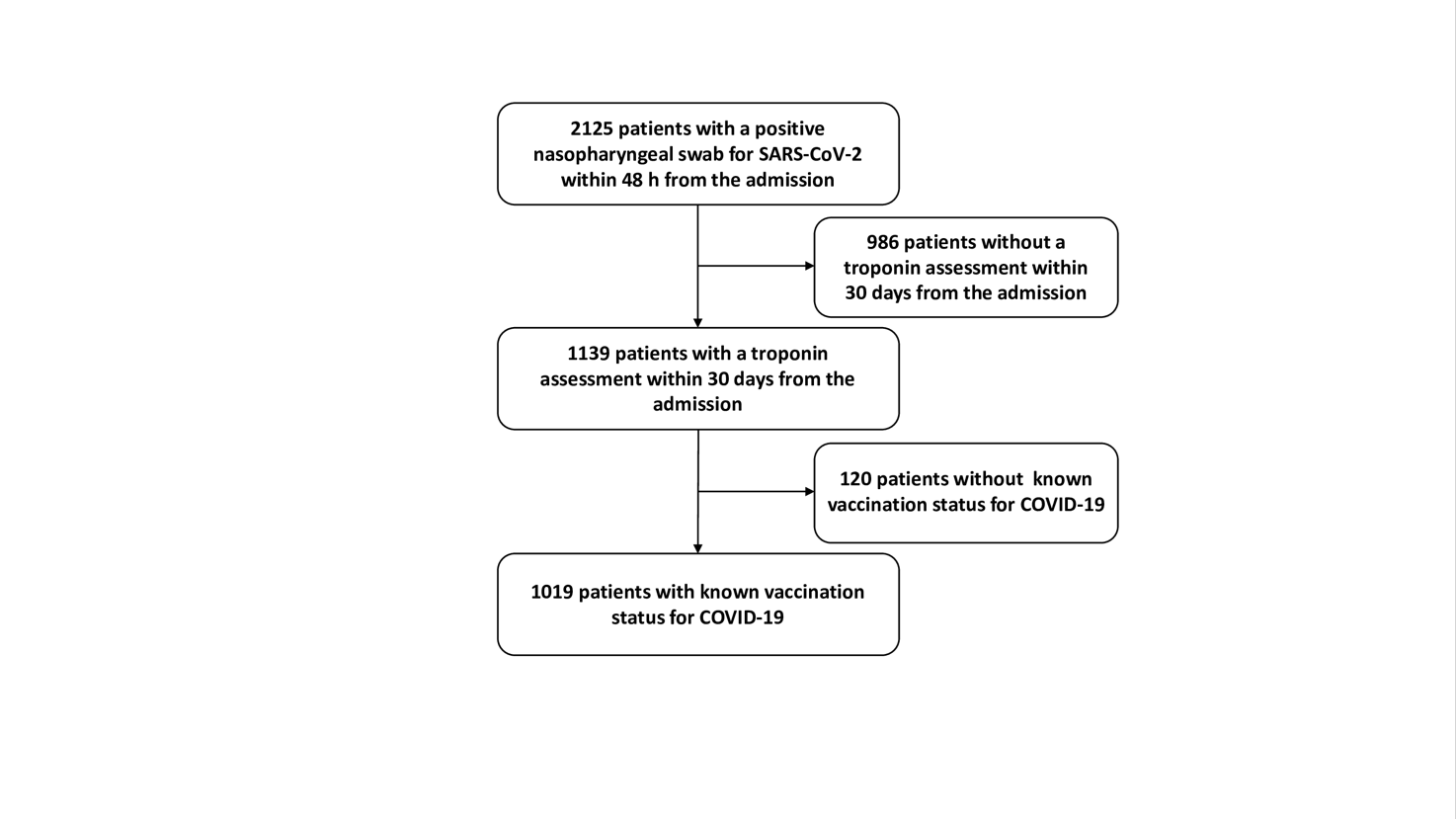
**

***Appendix Figure Legend***

**Appendix Figure 1:** Study flowchart. *Abbreviations:* SARS-CoV-2: severe acute respiratory syndrome coronavirus 2; COVID-19: Coronavirus Disease 2019.
